# An international survey of nutrition practices in adult patients receiving veno-venous ECMO

**DOI:** 10.1186/2197-425X-3-S1-A295

**Published:** 2015-10-01

**Authors:** DE Bear, J Haslam, L Camporota, M Shankar-Hari, NA Barrett

**Affiliations:** Department of Nutrition and Dietetics, Guy's and St Thomas' NHS Foundation Trust, London, United Kingdom; Department of Critical Care, Guy's and St Thomas' NHS Foundation Trust, London, United Kingdom

## Introduction

The use of veno-venous Extra-corporeal Membrane Oxygenation (vv-ECMO) in adults with severe respiratory failure is increasing. Nutrition whilst on vv-ECMO is challenging as inadequate feeding, mainly due to gastric intolerance is common [1–4]. Our understanding of how to meet nutrient needs in this high-risk patient is limited.

## Objectives

To improve our understanding of the nutritional support preferences and practices by conducting an international survey of adult ECMO centres.

## Methods

Details for International ECMO centres were obtained from our database used in previous surveys. Survey questions were developed through an iterative process and assessed for content and face validity. None of the survey domains were mandatory. This resulting 38-question online survey was emailed to 161 eligible ECMO centres using Smart Survey® (Smartline International Ltd, Gloucestershire, UK). Sole paediatric centres were excluded. The need for informed consent was waived by the local research and ethics committee.

## Results

Sixty-seven (42%) eligible centres responded to the survey. One third of centres managed less than 10 vv-ECMO patients over the previous year.

### Estimating Nutritional Requirements

The most common equation to determine energy requirements is 20-25kcal/kg/day (17/34; 50%) and 1.5g/kg/day for protein (11/32; 34%).

### Route of Initial Feeding

Out of 37 responses, 29 (78%) use naso-gastic feeding as the initial route of feeding. Five commence parenteral nutrition first (14%) and 3 (8%) post-pyloric feeding.

### Early Enteral Feeding

Twenty two out of thirty six respondents (61%) report feeding within 24 hours of commencing vv-ECMO. Twelve centres (33%) feed within 48 hours and one (3%) within 72 hours. One centre (3%) reports never feeding their vv-ECMO patients.

### Gastro-Intestinal Intolerance

Gastro-intestinal intolerance is perceived as common in these patients with 16 out of 26 respondents (69%) reporting that 50% or more of their patients require prokinetics drugs during their vv-ECMO run.

## Conclusions

The survey identifies significant heterogeneity in assessment and provision of nutritional requirement in these patients. Lack of evidence coupled with lack of guidelines relating to nutritional support explains this heterogeneity. Our survey highlights an urgent need for focused research in this area.

## References

[1] Scott LK (2004). Early enteral feedings in adults receiving venovenous ECMO. JPEN.

[2] Lukas G (2010). Nutritional support in adult patients receiving extracorporeal membrane oxygenation. Crit Care Resusc.

[3] Ferrie S (2013). Nutrition support during extracorporeal oxygenation (ECMO) in adults: a retrospective audit of 86 patients. ICM.

[4] Bear DE, et al: Jejunal feeding associated with improved nutrient delivery compared with gastric feeding in patients receiving extracorporeal membrane oxygenation. ICM. 2011, S294-Sept, Suppl 1

